# Gap solitons on an integrated CMOS chip

**DOI:** 10.1515/nanoph-2022-0623

**Published:** 2023-01-16

**Authors:** Ju Won Choi, Byoung-Uk Sohn, Ezgi Sahin, George F. R. Chen, Peng Xing, Doris K. T. Ng, Benjamin J. Eggleton, Dawn T. H. Tan

**Affiliations:** Photonics Devices and System Group, Singapore University of Technology and Design, 8 Somapah Rd, Singapore 487372, Singapore; Institute of Microelectronics, A*STAR, 2 Fusionopolis Way, #08-02, Innovis Tower, Singapore 138634, Singapore; School of Physics, Institute of Photonics and Optical Science, The University of Sydney, Sydney, New South Wales 2006, Australia; The University of Sydney Nano Institute (Sydney Nano), The University of Sydney, Sydney, New South Wales 2006, Australia

**Keywords:** Bragg grating, CMOS, gap soliton, ultra-silicon-rich nitride

## Abstract

Nonlinear propagation in periodic media has been studied for decades, yielding demonstrations of numerous phenomena including strong temporal compression and slow light generation. Gap solitons, that propagate at frequencies inside the stopband, have been observed in optical fibres but have been elusive in photonic chips. In this manuscript, we investigate nonlinear pulse propagation in a chip-based nonlinear Bragg grating at frequencies inside the stopband and observe clear, unequivocal signatures of gap soliton propagation, including slow light, intensity-dependent transmission, intensity-dependent temporal delay and gap soliton compression. Our experiments which are performed in an on-chip ultra-silicon-rich nitride (USRN) Bragg grating with picosecond time scales, reveal slow light group velocity reduction to 35%–40% of the speed of light in vacuum, change in the temporal delay of 7 ps at low peak powers between 15.7 W–36.6 W, which is accompanied by up to 2.7× temporal compression of input pulses. Theoretical calculations using the nonlinear coupled mode equations confirm the observations of intensity-dependent temporal delay. Of fundamental importance, this demonstration opens up on-chip platforms for novel experimental studies of gap solitons as the basis of all-optical buffers, delay lines and optical storage.

## 1 Introduction

Bragg solitons are classified as solitary waves which form in periodic media, facilitated by the group velocity dispersion induced by the medium’s periodicity and its nonlinearity. The first theoretical predictions of Bragg solitons was in 1989 [1, 2] and they were observed in 1996 [3] in optical fibre Bragg gratings, revealing pulse compression and slow light. This demonstration and the related theoretical frameworks paved the way for further details studies of Bragg soliton phenomena [4–8], with experimental efforts focused on Bragg gratings incorporated into silica-based optical fibres. These experiments leveraged the developments of fibre Bragg gratings for the telecom industry and the low-loss of silica-based optical fibres but required high peak powers due to the modest optical nonlinearity of silica.

Gap solitons are a subset of Bragg solitons, possessing important, distinctive identifying features – They are generated when Bragg soliton states exist *within the forbidden stopband* of periodic structures [9, 10] and are linked to intensity-dependent transmission (self-switching) as well as the soliton dynamics and slow light associated with the more general class of Bragg solitons. In 1998, Taverner et al. reported experimental observations of multiple gap solitons optical self-switching in fibre Bragg gratings [11]. In 2006, Mok et al., reported observations of transmitted and reflected gap solitons, and the group velocity of the gap soliton was documented to reach 16% of the speed of light in vacuum [12]. From these experimental and related theoretical studies, the key signatures linked to gap solitons are: (i) intensity-dependent transmission associated with the self-switching of the stopband, (ii) soliton dynamics such as pulse compression and fission and (iii) slow light behaviour. Beyond these three signature characteristics, the reflected component of gap solitons can possess a recess in the pulse [12], which arises due to the temporal dynamics of the intensity dependence intrinsic to gap soliton formation.

Recently, Bragg soliton phenomena on a chip were demonstrated for the first time, with the observation of Bragg soliton effect compression and soliton fission in a compact, highly nonlinear platform using modest peak powers [13–15]. The realization of gap solitons in chip-based platforms is however non trivial relative to the fibre demonstration. In particular, the compact nature of integrated on-chip platforms, where device lengths are often limited to the centimeter scale or less, requires strong nonlinear phase shifts. Furthermore, the formation of gap soliton states with solutions residing *within the photonic bandgap* requires a high-quality Bragg grating with sharp band edges which allows the optical field to switch into the slow light regime at the band edge. The demands on the Bragg grating are compounded because the shorter grating length necessitates a large coupling coefficient to ensure strong extinction. Since the bandwidth of the grating scales with the coupling coefficient, the shorter waveguide length results in a higher optical intensity requirement for the observation of gap solitons. In light of these difficulties, the ability to create high quality on-chip Bragg gratings with well-defined stopbands possessing sharp edges, as well as minimal transmission or phase ripple is paramount [16–19].

On-chip Bragg gratings on a platform with high nonlinearity and negligible two-photon absorption could further allow gap soliton formation at relatively low powers, facilitating the observation of intensity-dependent transmission, soliton dynamics and slow light behaviour, the key characteristics of gap solitons.

In this paper, we present experimental observations of gap soliton formation using high-quality on-chip Bragg gratings implemented on ultra-silicon-rich nitride (USRN) [13, 14, 20, 21]. The dramatic variation in group index/transmission at the stopband edges is enabled by the cladding-based modulation which overcomes on-chip fabrication challenges in achieving gratings possessing high quality transmission properties with sharp band edges [13], facilitating the observation of gap soliton induced tunable slow light. We experimentally observe the gap soliton propagation including slow light, temporal soliton compression, and the tuning of intensity-dependent temporal delay, with good agreement with theory achieved. The intensity-dependent Kerr effect induces varying extents of red-shifts in the stopband, which can be observed by intensity-dependent transmission of the grating. The intensity-dependent transmission induces the intensity-dependent temporal delay resulting in deceleration of the gap soliton close to the photonic bandgap [12]. The change in the temporal delay of 7 ps is experimentally achieved at a low peak power of 15.7 W–36.6 W, which shows good agreement with theoretical calculations using the nonlinear coupled mode equation. Harnessing the measured group index, the maximum slow light experimentally achieved is 80% of the group velocity of a USRN waveguide, equivalent to 35% of the phase velocity of light in vacuum at a peak power of 15.7 W. We further document an associated 2.7× temporal soliton compression of the optical pulses, and theoretically analyze the impact of the grating length on the gap soliton dynamics. This work showcases the advantages of gap solitons towards potential slow light applications, including optical delay lines and optical storage in a compact, CMOS-compatible chip.

## 2 Results and discussion

### 2.1 The principle of the bandgap shift and optical properties of USRN CMBG

**Figure 1:**
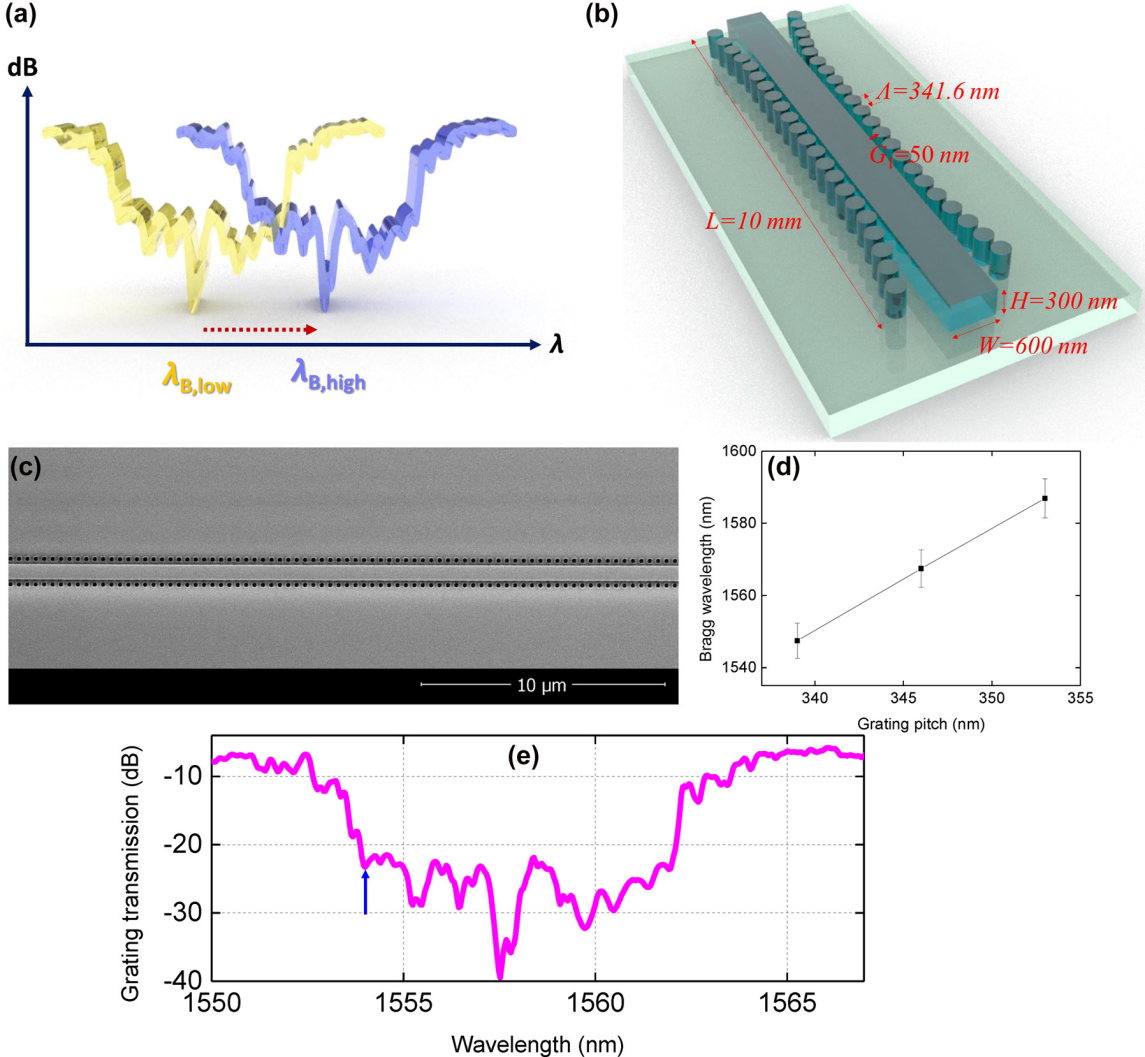
Principles of Kerr-based gap soliton formation and optical properties of the USRN CMBG. (a) The spectral shift of the photonic bandgap when subjected to high and low input intensity (Blue line: High intensity, Yellow Line: Low intensity. (b) The schematic of the USRN CMBG. (c) A scanning electron micrograph of the CMBG. (d) Measured Bragg wavelength (*λ*_B_) as a function of grating pitch (Λ) as well as statistical error bars for five gratings of identical pitch for each value of grating pitch. (e) Linear grating transmission of the USRN CMBG. The blue arrow denotes the wavelength of the pump.

The principle of the formation of gap solitons arising from a Kerr-induced bandgap shift, as well as the optical properties of USRN Bragg grating (cladding modulated Bragg gratings (CMBG)) used for this work are shown in Figure 1. The Bragg grating utilizes a cladding-modulated configuration, where cylinders are placed alongside the central waveguide periodically, so as to create the effective index modulation. The gap between the cylinders and the central waveguide varies in a raised cosine profile so as to introduce an effective apodization.

When light of intensity, *I* propagates through the CMBG, the refractive index, *n* of the medium experiences an increase from the Kerr effect [22] according to the equation, *n* = *n*_0_ + *n*_2_ *I*, where *n*_0_ is the refractive index at low intensities, and *n*_2_ is the nonlinear refractive index. *I* is the input laser’s intensity, given by $\frac{P_{\text{in}}}{A_{\text{eff}}}$ , where *P*_in_ and *A*_eff_ are the input peak power and effective mode area, respectively. Consequently, a sufficiently large intensity of light can generate a red-shift of the grating’s photonic bandgap, as shown in Figure 1(a) [12]. The index difference between high and low input intensities is represented by Δ*n* = *n*_2_ *I*. The period of a Bragg grating (Λ) is defined by $\frac{\lambda_{B}}{2n}$ , where *λ*_B_ is the Bragg wavelength depicting the central wavelength of the photonic bandgap. The difference between the Bragg wavelength at low and high peak powers (*λ*_B,low_ and *λ*_B,high_) is further given by,

(1) $$\Delta \lambda_{B}=2\Delta n\Lambda =2n_{2}I\Lambda =\frac{2n_{2}\Lambda P_{\text{in}}}{A_{\text{eff}}}$$

Equation (1) indicates that the extent of red shift in the Bragg wavelength increases for high input peak power. Based on this principle, we can achieve power-dependent slow light tuning when the wavelength of incident optical pulses is positioned at the photonic band edge: Increasing the incident optical intensity will result in a greater red-shift of the photonic bandgap, concomitantly resulting in a variation in the group velocity from 0 to *υ*_g_ (*υ*_g_ = group velocity without the Bragg grating effect) [23, 24].

Δ*n* required for a sufficiently large group velocity change is facilitated by a large nonlinear refractive index and small effective area. According to Equation (1), both parameters facilitate a red-shift of the photonic bandgap. Integrated waveguides with high refractive index contrast between core and cladding are prime candidates for implementing small *A*_eff_. The USRN platform is designed to have a sufficiently large band gap eliminating two-photon absorption (TPA) while having a large nonlinear refractive index at the 1550 nm wavelength. The high silicon content in USRN is achieved by using a higher SiH_4_:N_2_ precursor gas ratio during chemical vapor deposition compared to that used in stoichiometric silicon nitride. We have previously characterized the film’s optical properties using Fourier transform infrared spectroscopy (FTIR) and X-ray photoelectron spectroscopy (XPS) to determine *n*–*k* values and the atomic composition of silicon (Si) and nitrogen (N)/information of the energy bandgap, respectively. *n*–*k* values from FTIR measurement show our film possesses a large linear refractive index of 3.1 at 1550 nm with a bandgap of 2.1 eV having a negligible TPA at the atomic composition of Si:N = 7:3, which is measured by XPS. Miller’s rule indicates that the film possessing a larger linear refractive index has a larger nonlinear refractive index. The Kerr nonlinear refractive index was previously characterized by the measurement of self-phase modulation and the achieved nonlinear parameters. The strong achieved nonlinearity, *n*_2_ = 2.8 × 10^−13^ cm^2^/W, which is 2 orders of magnitude larger than that in stoichiometric silicon nitride, [20, 25, 26], was quantified using both z-scan measurements of the bulk material [21], as well as self-phase modulation experiments [25]. This platform is therefore selected for the implementation of on-chip Bragg gratings for observations of gap soliton phenomena.

Fabrication of the USRN CMBGs was performed by first depositing a 300-nm-thick USRN film on a thermal oxide layer on a silicon substrate using inductively coupled chemical vapor deposition at a temperature of 250 °C. Patterning of the structures was performed using electron-beam lithography followed by inductively coupled reactive ion etching, and plasma-enhanced chemical vapor deposition of the SiO_2_ overcladding.

The photonic bandgap is implemented using a CMBG structure, which results in a large magnitude of group velocity dispersion (GVD). CMBGs provide an effective refractive index modulation from cylindrical pillars placed alongside a central waveguide, with control of the strength of the refractive index modulation achieved by varying the distance to the central waveguide. Consequently, apodization necessary to achieve smooth phase and magnitude spectra can be easily implemented [13, 14].

We implement the CMBGs using a USRN core (*W* = 600 nm by *H* = 300 nm) with SiO_2_ under- and over-cladding. Figure 1(b) shows a schematic of the CMBG. The various CMBG parameters are the pillar radius of 100 nm, grating pitch (Λ) of 341.6 nm, and gap distance (*G*_1_) between the central waveguide to the pillar at the middle of the CMBGs, selected to be 50 nm, respectively. The total CMBG length (*L*) is 10 mm. The scanning micrograph image is shown in Figure 1(c).

Figure 1(d) shows the fabrication reproducibility by measuring the variation of *λ*_B_ at each Λ. We fabricate five gratings with the same Λ and the experimentally measured *λ*_B_ (together with the statistical error bars) are plotted in the figure. For each CMBG with the same designed Λ, the *λ*_B_ is obtained to have 4–5 nm of variation. Importantly, the *λ*_B_ for different grating pitches can be discriminated from one another, even though the difference in the pitch is less than 10 nm (7 nm). Based on the Bragg condition which governs *λ*_B_ = 2*n*_eff_Λ where *n*_eff_ is the effective index of the waveguide, the *λ*_B_ is proportional to the Λ. The figure below shows that the fabrication is sufficiently accurate to distinguish between CMBGs with a pitch difference of 7 nm. In the future, achieving the exact *λ*_B_ may also be facilitated by utilizing thermo-optic tuning.

The linear grating transmission of the fabricated CMBG is shown in Figure 1(e). The central wavelength of the bandgap is 1557.8 nm with a bandwidth of 10 nm. The propagation loss of the CMBG outside the photonic bandgap region is 6.6 dB/cm. We choose 1554 nm as a pump wavelength as denoted with the blue arrow of Figure 1(e) because transmission is expected to increase as the input power increases due to the red-shift of the photonic bandgap. The ratio of the stopband bandwidth to the laser bandwidth is 25, indicating that the laser bandwidth is narrow enough to observe the variation of output power as a result of the shift in the bandgap.

### 2.2 Theoretical analysis of gap soliton generation

(2) $$\begin{array}{ll} & +i\frac{\partial A_{+}}{\partial z}+i\frac{n}{c}\frac{\partial A_{+}}{\partial t}+\delta A_{+}+\kappa A_{-}+\gamma \left({\left|A_{+}\right|}^{2}+2{\left|A_{-}\right|}^{2}\right)\\ & \;\times A_{+}=0, \end{array}$$

$$\begin{array}{ll} & -\frac{\partial A_{-}}{\partial z}+i\frac{n}{c}\frac{\partial A_{-}}{\partial t}+\delta A_{-}+\kappa A_{+}+\gamma \left({\left|A_{-}\right|}^{2}+2{\left|A_{+}\right|}^{2}\right)\\ & \;\times A_{-}=0, \end{array}$$

The gap soliton dynamics are analyzed using the nonlinear coupled mode equations as shown in Equation (2) describing the pulse propagation through the CMBG [12, 27]. *A*_+_ and *A*_−_ are the electric-field envelopes of the forward and backward propagating modes, respectively. In our simulations, we model the forward propagating modes in order to corroborate our experimental observations. The pulse wave-Bragg detuning (*δ*), the grating coupling strength (*κ*), nonlinear parameter (*γ*), and *n* are calculated based on the optical properties of the CMBG as shown in Figure 1(b) and (e). The pulse wavelength-Bragg detuning is denoted by $\delta =2\pi n\left(\frac{1}{\lambda }-\frac{1}{\lambda_{B}}\right)$ , where *λ* is the input wavelength. The *δ* is varied by *n* and *λ*_B_ which depend on input peak power. The grating’s coupling strength is defined as $\kappa =\frac{\pi \cdot \Delta n_{a}}{\lambda_{B}}$ , and is estimated to be 21,900 m^−1^ by matching the edge of the bandgap (grating transmission = −18.2 dB as shown in Figure 1(e)), where *κ* = *δ*Δ*n*_*a*_ is the amplitude modulation of the linear refractive index. Δ*n*_*a*_ is the amplitude of the index modulation. The strong grating dispersion can be derived from the linear coupling, which is proportional to *κ* in the nonlinear coupled mode equation. As a consequence, *κ* represents the grating dispersion term as no dispersion term is included in the coupled mode equation [12, 28]. The nonlinear parameter, *γ* = 435 W^−1^/m. 

**Figure 2:**
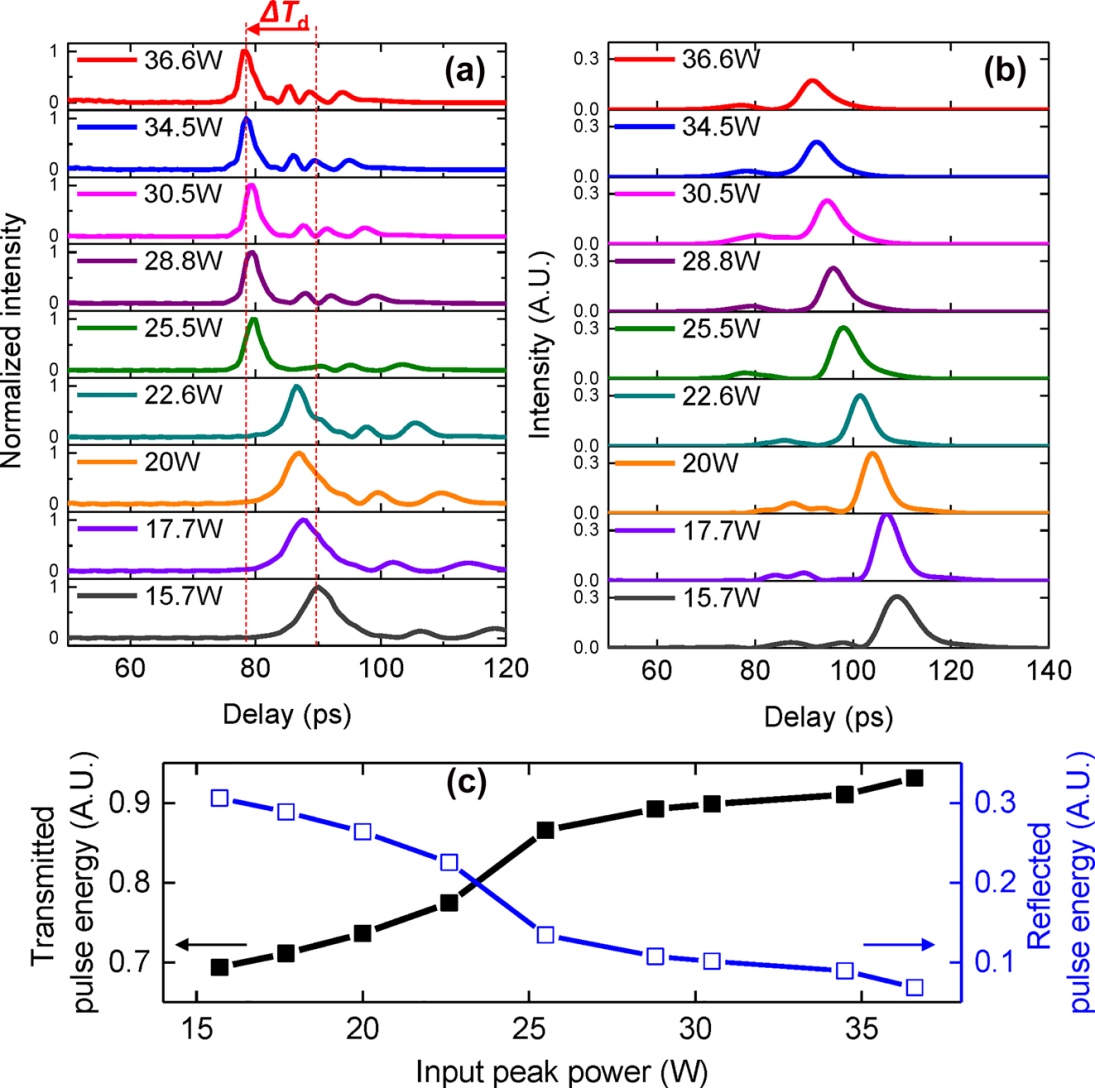
Numerically calculated evolution of temporal traces for transmitted (a) and reflected pulses (b) as input peak power of the pulses launched into the 10 mm CMBG is varied. (c) Integrating pulse energies of transmitted and reflected as a function of input peak power. The total pulse energy for each input peak power is fixed at 1. The input pulse width is 9 ps.

**Figure 3:**
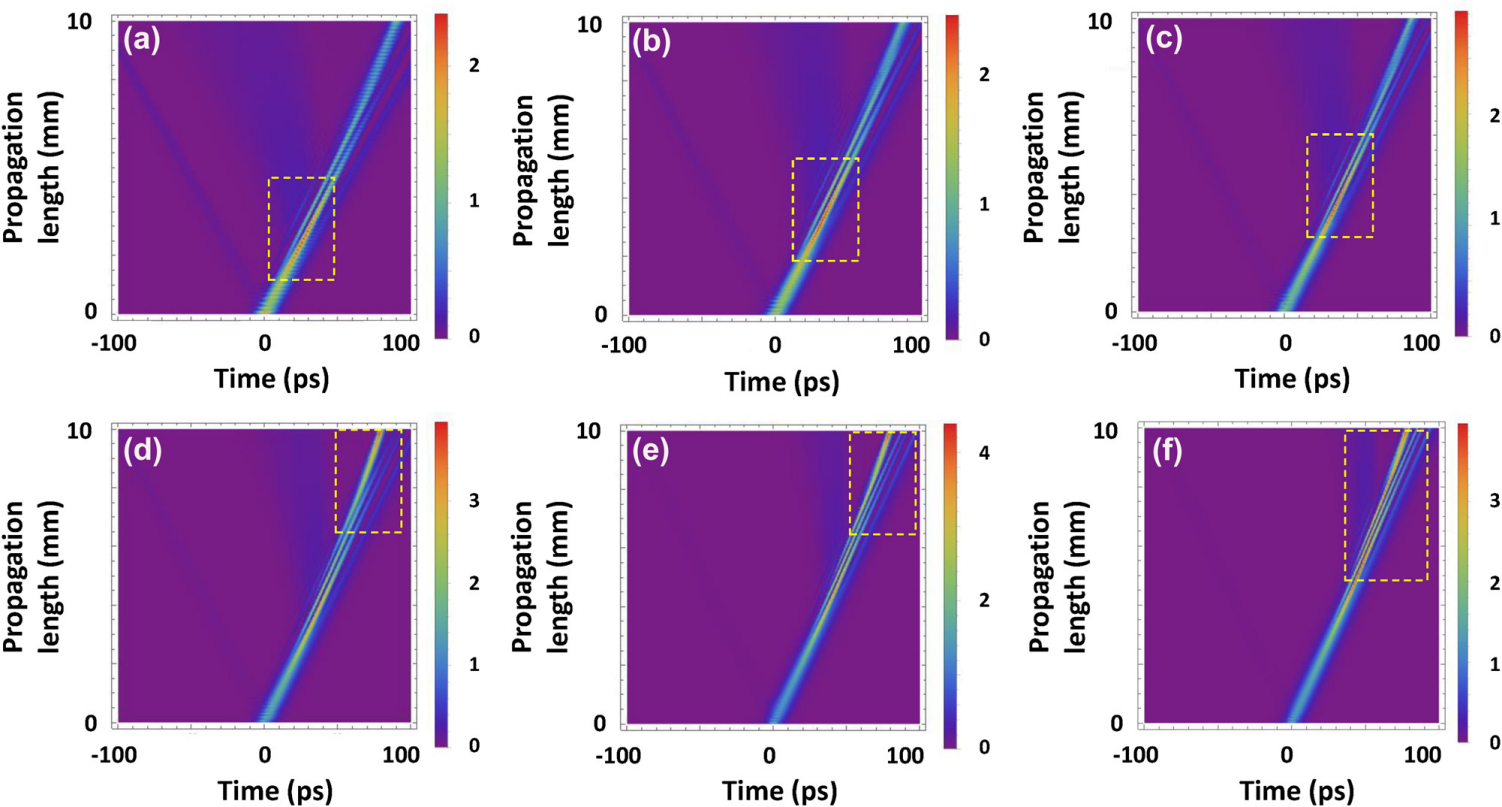
The evolution of the gap soliton as it propagates through the CMBG for *P*_in_ = 15.7 W (a), *P*_in_ = 20 W (b), *P*_in_ = 22.6 W (c), *P*_in_ = 25.5 W (d), *P*_in_ = 30.5 W (e), and *P*_in_ = 36.6 W (f). The yellow boxes depict the location where maximum intensity is achieved along propagation.

The theoretical gap soliton dynamics as the pulse propagates through the 10 mm CMBG are observed as the input peak power is varied as shown in Figure 2. In the simulations, the 9 ps input pulses are located at *t* = 0 at the CMBG input. The output waveform at the end of CMBG propagation is numerically calculated as a function of input peak power as shown in Figure 2(a). The temporal delay is observed to decrease as *P*_in_ is increased from 15.7 W to 36.6 W, consistent with the nature of gap solitons. Steep variations in the peak value are shown when *P*_in_ changes from 22.6 W to 25.5 W where optical soliton condition is satisfied in simulation. The change in temporal delay (Δ*T*_d_) between *P*_in_ = 15.7 and 36.6 W is observed to be 12 ps. In addition, the output pulses are observed to experience compression, with the pulse at *P*_in_ = 36.6 W being compressed by a factor of 2.7 × to 3.3 ps. The intensity and number of sidelobes increases because more solitons are generated as *P*_in_ increases. The temporal waveform of the reflected pulses may be used as an indicator of the gap soliton effect [12]. As the pulses’ temporal width is 10× shorter than the temporal delay, the truncation of transmitted solitons in the reflected pulses is not easily observed. However, Figure 2(b) shows a small recess near the fast-moving part of reflected pulses, which results in an asymmetry in the waveforms. The truncated parts indicate where gap-soliton waveforms are created in transmission. The intensity of reflected pulses decreases as *P*_in_ increases because more soliton states are generated at higher power as shown in Figure 2(c) (blue empty squares). Conversely, the intensity of transmitted pulses increases as *P*_in_ increases as shown in Figure 2(c) (black solid squares). Correspondingly, the transmission of more input energy goes towards the formation of the gap soliton, resulting in less energy possessed by the reflected pulses as *P*_in_ increases. The evolution of gap solitons is observed as pulses propagate through the CMBG and *P*_in_ is varied from 15.7 W to 36.6 W as shown in Figure 3. The yellow dashed boxes depict the location where maximum pulse intensity is achieved along the propagation direction. In Figure 3(a)–(c), the maximum intensity is observed mid-propagation onwards as *P*_in_ increases from 15.7 W to 22.6 W, but the intensity is weakened at the end of the propagation. At *P*_in_ of 25.5 W, more solitons start to be generated due to the higher power by splitting the pulses into one main peak and sidebands as shown in Figure 3(d). The creation of sidebands enables us to achieve the compressed main pulse. Consequently, this leads to the strong intensity becoming visible at the end of propagation in the grating. Therefore, the temporal position where maximum intensity is located at the output varies steeply between 22.6 W and 25.5 W. For *P*_in_ >25.5 W as shown in Figure 3(e) and (f), stronger temporal compression is achieved with sidebands enhancement as more solitons are generated. The pulses are observed to encounter a temporal delay as they propagate through the CMBG while simultaneously developing sidebands. The extent of temporal shift over the CMBG depends on the change in refractive index distribution, which in turn provides varying extents of temporal retardation, representative of slow light generation.

### 2.3 Experimental characterization of gap soliton propagation

The gap soliton propagation is demonstrated experimentally. 9 ps pulses from a fibre laser, amplified by an erbium-doped fibre amplifier, is used as a pump. The power of the pulses is varied using a digital attenuator. The amplified input pulses pass through a circulator. An oscilloscope is used to measure the temporal delays experienced by pulses at the CMBG output. The oscilloscope bandwidth is 40 GHz. Consequently, the temporal resolution is limited to around 18 ps and the exact temporal waveforms/pulse widths of the output cannot be observed, particularly when considering the input pulse duration of 9 ps. However, the oscilloscope is able to experimentally characterize the change in temporal delays experienced by the pulses as a result of slow light effects associated with the gap soliton. We expect to capture a single waveform using the oscilloscope, which can be used to determine the temporal location of each pulse as a function of optical power.

**Figure 4:**
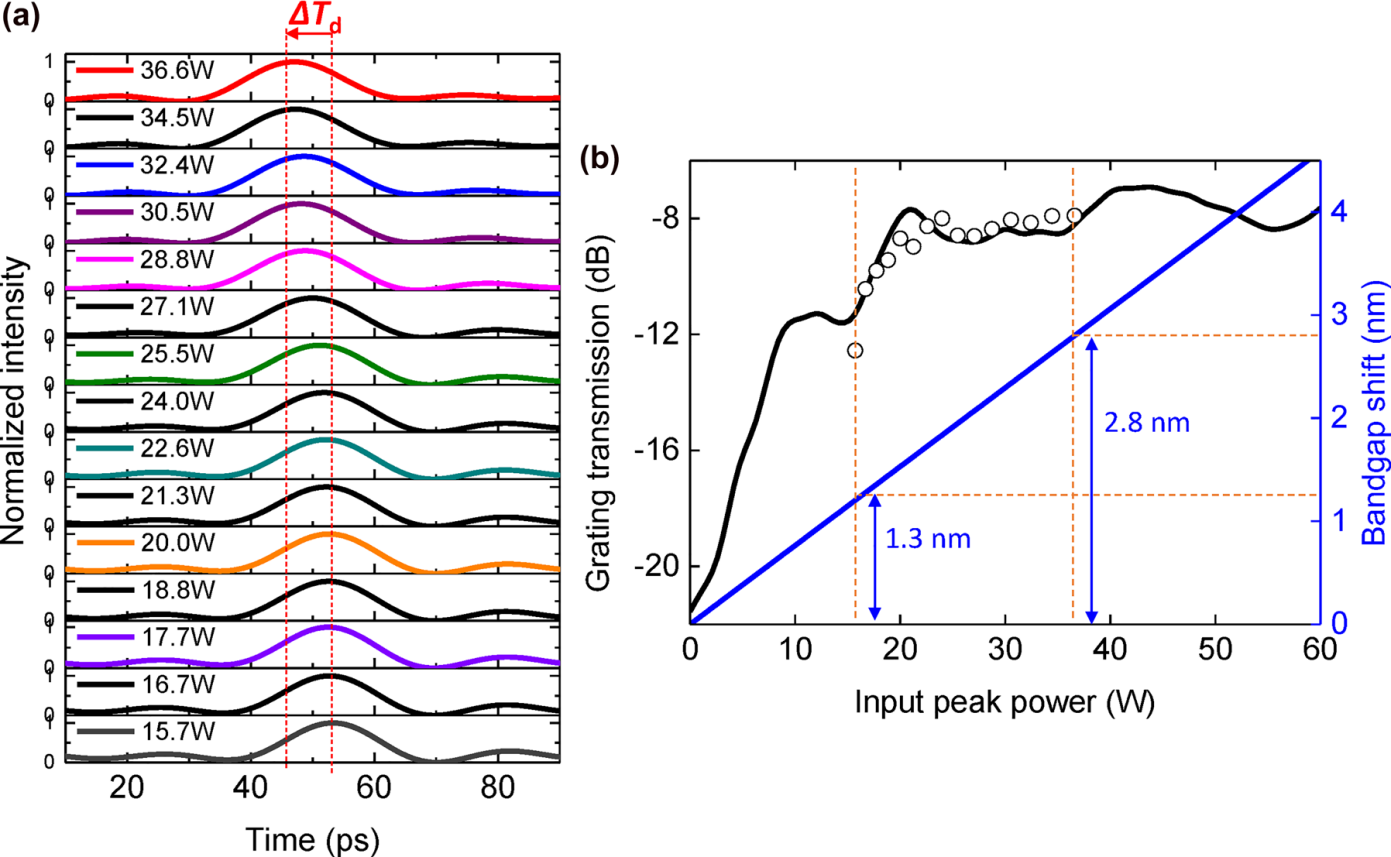
Experimental characterization of temporal delay and transmission in the CMBG. (a) Experimentally measured temporal delay variation of optical pulses as a function of input peak power. (b) The measured output power to input power (dB) of the pulses as the input peak power is varied (empty circle dots). This is compared with the linear grating transmission spectrum of the CMBG. The blue line denotes the Bandgap shift.

Temporal waveforms are measured as the input peak power of the pulses is varied from 15.7 W to 36.6 W as shown in Figure 4(a). We observe a decrease in the temporal delay (Δ*T*_d_) as the input power is increased, which follows the theoretical trend. This effect originates from the group velocity retardation becoming less pronounced when *P*_in_ increases, resulting in a smaller temporal delay in the output. The measured Δ*T*_d_ for *P*_in_ = 15.7 W–36.6 W is 7 ps. We note that the absolute value of the temporal location in each waveform is quantified with respect to the optical path length induced by the trigger line on the measurement system. The variation of output power to input power (dB) after passing the CMBG is observed as shown in Figure 4(b). The grating transmission as a function of wavelength as shown in Figure 1(e) is plotted in terms of *P*_in_ (black line in Figure 4(b)) using Equation (1) for comparison with the measured transmission (empty circle). The measured transmission (empty circle) is increased as *P*_in_ increases which corresponds with the transmission (black line). The bandgap shift can be calculated using Equation (1), corresponding with a maximum bandgap shift of 2.8 nm for *P*_in_ = 36.6 W which indicates the Bragg wavelength has shifted to 1560.55 nm. It shows that the 5 dB variation in the measured transmission (empty circle) is consistent with the calculated bandgap shift of 1.5 nm at *P*_in_ of 15.7 W–36.6 W. Note that, waveforms for *P*_in_ < 15.7 W were not measurable in our experimental setup due to its close proximity to the center of the bandgap, arising in the low transmission and low output power.

We note further that self-phase modulation (SPM) induced spectral broadening can also result in an intensity-dependent transmission similar to that caused by the gap soliton effect. A condition was previously established in Reference [4] that the nonlinear phase shift of the pulse preceding its propagation in the Bragg grating should not exceed 0.5π in order to ensure that SPM’s contribution to any observed intensity-dependent transmission is negligible. In our devices, inverse tapers to facilitate fibre-waveguide coupling with a length of 200 μm precede the CMBG. The input taper, which input pulses encounter first, induces a nonlinear phase shift that does not exceed 0.5π. Therefore, the increased transmission as shown in Figure 4(b) is a result of the gap-soliton effect and not SPM [4].

### 2.4 Gap soliton-based slow light

**Figure 5:**
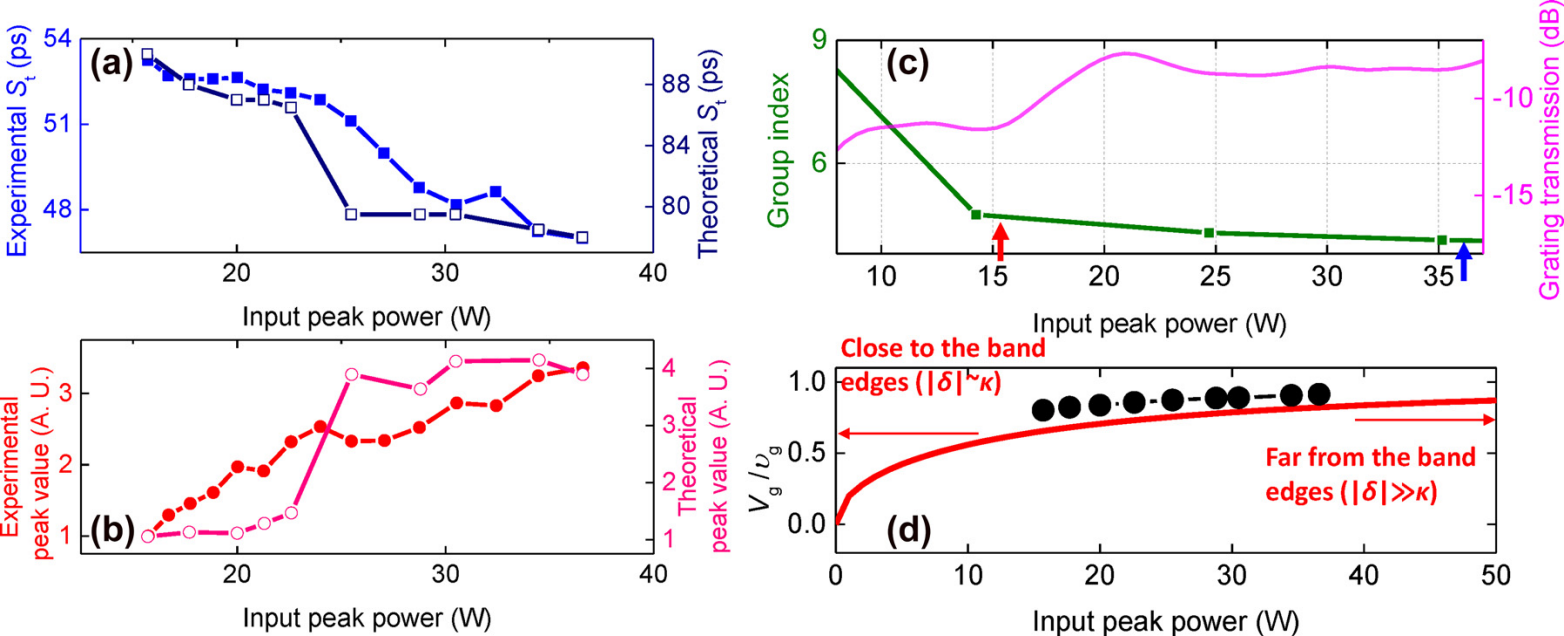
The experimentally measured (solid dots) and simulated (empty dots) variation of temporal position, *S*_t_ (a) and peak value (b) as a function of input peak power. (c) The experimentally measured group index (green line) and transmission (fuchsia line) near the photonic bandgap. Red and blue arrows depict the group indices at *P*_in_ = 15.7 W and *P*_in_ = 36.6 W, respectively. (d) Ratio of group velocity of the CMBG (*V*_g_) to the group velocity far from the grating stopband (*υ*_g_) as a function of input peak power (theory (−), experiment (●)).

Next, we investigate the temporal delay of the pulses as a function of peak power and its agreement with theory. Figure 5(a) plots *S*_t_, the experimentally measured location of the pulse center corresponding to Figure 4(a) together with the theoretically calculated value corresponding to Figure 2(a). It may be observed that the experimental trend as a function of the input peak power agrees well with the theoretically calculated value. Figure 5(b) further plots the experimental peak value (amplitude of the pulse center) corresponding to Figure 4(a), where the trend showing an overall increase in the peak value as a function of input peak power agrees well with the theoretically calculated values corresponding to Figure 2(a). We obtain a 3.4× and 3.9× increase in the peak value from the experiment and simulation respectively when varying *P*_in_ between 15.7 W and 36.6 W as shown in Figure 5(b). The group velocity is defined as $\frac{L}{T_{d}}=\frac{c}{n_{g}}$ , where *L*, *T*_d_, *c* and *n*_g_ are the CMBG length, temporal delay, speed of light in vacuum and group index respectively. Therefore, the group index change (Δ*n*_g_) can be defined as $\frac{c\Delta T_{d}}{\Delta L}$ , where Δ*T*_d_ is the change in temporal delay and Δ*L* is the propagation length. The variation in the temporal position as a function of *P*_in_ shown in Figure 5(a) provides the change in temporal delay as indicated above. We obtain a Δ*T*_d_ of 7 ps and 12 ps in the experiment and simulation respectively. This corresponds to Δ*n*_g_ of 0.2 and 0.3 from experiments and simulations respectively, showing the values are fairly close each other. Steep variations in the temporal variation and peak value are observed in simulations when *P*_in_ changes from 22.6 W to 25.5 W. In this range of peak powers, conditions for soliton formation are satisfied. The discrepancy between Δ*T*_d_ in the experiment and simulation originates from the limited resolution of the oscilloscope in the experiment. Theoretical temporal traces have one main peak and sidebands at higher power, whereas the experimentally measured temporal waveforms are broader and symmetric because they can only present the averaged profile within the temporal resolution of >18 ps, which is larger than that of theoretical observation. It indicates that *S*_t_ of the experimental waveform moves back to a longer temporal delay than that in theory at the higher power leading to Δ*T*_d_ in the experiment being shorter than that in theory. For the temporal traces at *P*_in_ = 36.6 W, the averaged *S*_t_ among sidebands is located at around 89 ps. Those sidebands possess 34% of the total integrating temporal intensity. It means that the temporal delay in the averaged waveform is observed to be located 3.74 ps longer than the main peak’s temporal delay in theory. It indicates Δ*T*_d_ ∼ 8 ps is observed in the averaged waveform which is quite close to the experimentally measured Δ*T*_d_ = 7 ps using the oscilloscope. In addition, the overall trend reflecting an increase in the peak value as a function of peak power is clear from both the experiments and theory.

We can further observe whether the group velocity of pulses is slower in the CMBG compared to that without the grating effect. The group index can be estimated as the experimental measurement gives the bandgap shift at each peak power. Figure 5(c) shows the measured group index of the CMBG (green line), measured using an interferometric component analyzer. Based on the measured group index, we can also extract *V*_g_/*υ*_g_ which is the ratio of group velocity of the CMBGs to the group velocity far from the stopband as shown in the solid circles of Figure 5(d). *V*_g_/*υ*_g_ is 0.80 and 0.91 for *P*_in_ of 15.7 W and 36.6 W respectively, indicating that group velocity in the Bragg grating is reduced by 20% and 9% of *υ*_g_. *υ*_g_ can goes up to *c*/*n* as gap soliton can possess the velocity between 0 and *c*/*n* [27]. Consequently, this value of *V*_g_ corresponds to 35% and 40% of the speed of light in vacuum at *υ*_g_ = *c*/*n*, respectively.

The experimental result is compared with theory from the linear dispersion relation with the Bragg grating presented as *q* ^2^ = *δ* ^2^ − *κ* ^2^, where *q* is shown as *k* − *k*_*B*_. (*k* = wavenumber, *k*_B_ = wavenumber at Bragg condition) Therefore, the group velocity of light propagating in the Bragg grating can be found from the dispersion relation as $V_{g}=\pm \upsilon_{g}{\left(1-\frac{\kappa^{2}}{\delta^{2}}\right)}^{\frac{1}{2}}$ , where *V*_g_ is group velocity when sufficiently close to the grating stopband and therefore subject to a grating induced slow light effect. *υ*_g_ refers to the group velocity in absence of and grating-induced effect [29]. ± refer to the forward/backward propagating pulses. We can estimate *δ* given a specific input power and fixed value of *κ*. Far from the band edges (|*δ*|>>*κ*), *V*_g_ is close to *υ*_g_ (*V*_g_/*υ*_g_ ∼ 1). Close to the photonic stopband (|*δ*| approaches *κ*), an optical pulse experiences a slowing effect from the presence of the grating. In the limit where the pulse wavelength is tuned closer and closer to the photonic bandgap, *V*_g_ approaches 0. *V*_g_/*υ*_g_ is 0.65 and 0.82 for *P*_in_ of 15.7 W and 36.6 W respectively, indicating that group velocity in the Bragg grating is reduced by 35% and 18% of *υ*_g_ as shown in the red line of Figure 5(d). This value of *V*_g_ corresponds to 28% and 36% of the speed of light in vacuum at *υ*_g_ = *c*/*n*, respectively.

It may be observed from Figure 5(d) that the trend of experimental group velocity as a function of input peak power agrees well with the theoretically obtained values. In both cases, a higher input peak power leads to a monotonic decrease in the group velocity. Comparing *P*_in_ = 15.7 W and *P*_in_ = 36.6 W, Δ*n*_g_ based on the measured group index is 0.5 which is close to the value of Δ*n*_g_ ∼ 0.3 obtained from the temporal shift measured using the oscilloscope and the value determined from simulations.

### 2.5 Temporal compression of gap solitons

**Figure 6:**
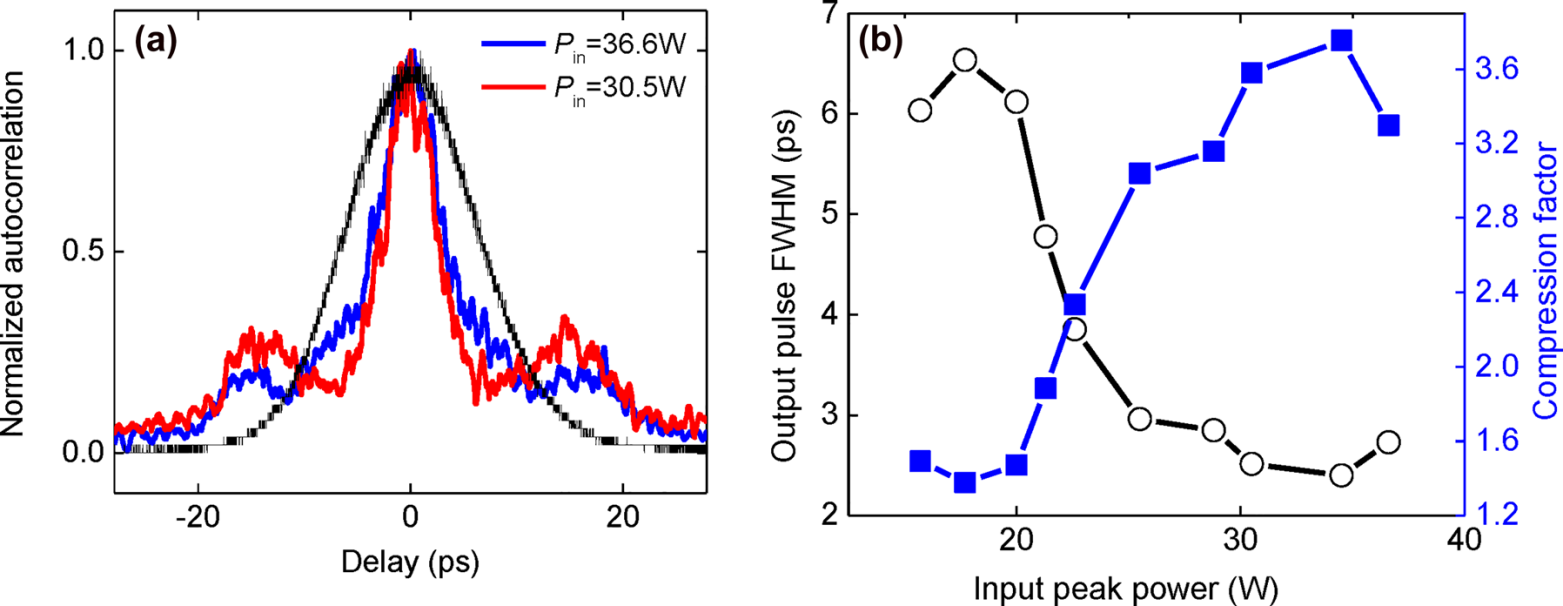
Temporal compression of gap solitons. (a) Experimentally measured, normalized autocorrelation traces for *P*_in_ = 30.5 W (red line) and *P*_in_ = 36.6 W (blue line). The black curve depicts the autocorrelation traces of input pulses. (b) Simulated output pulse full-width-half-maximum (FWHM) and compression factor as a function of input peak power.

In addition to the ability to slow light, gap solitons may also induce a temporal compression effect. We measure the temporal profile of the optical pulses propagating through the CMBG for *P*_in_ = 15.7 W–36.6 W using an autocorrelator. Figure 2(a) shows that the temporal compression strengthens as *P*_in_ increases. The magnitude of anomalous GVD increases near the blue band edge of the photonic bandgap where a concomitant decrease in linear transmission occurs. Therefore, strong temporal compression occurs where the pump is located near the blue-edge with high transmitted power. We observe autocorrelation traces at *P*_in_ of 30.5 W (red line) and 36.6 W (blue line) as shown in Figure 6(a). The pulse full-width-half-maximum (FWHM) is obtained by applying the deconvolution factor (1.54 for sech^2^ pulses, which has the temporal intensity pulse profile with the shape of a sech^2^ function) to the autocorrelation trace width [30]. The output pulse FWHM is 3.3 ps and 4.5 ps for *P*_in_ of 30.5 W and 36.6 W, indicating 2.7× and 2× compression respectively. The simulated pulse FWHM is obtained from Figure 2(a) and plotted as shown in Figure 6(b). The output FWHM is 2.5 ps and 2.7 ps for *P*_in_ of 30.5 W and 36.6 W, indicating 3.6× and 3.3× compression respectively in simulation. The measured experimental pulse widths are smaller than those in the simulation because the temporal delay is estimated to be longer in the simulation than in the experiment. However, the trend where compression is much higher for *P*_in_ = 30.5 W than for *P*_in_ = 36.6 W is consistent for both the simulation and experiment. This is because *P*_in_ of 30.5 W to is more conducive for temporal compression.

### 2.6 Discussion

**Figure 7:**
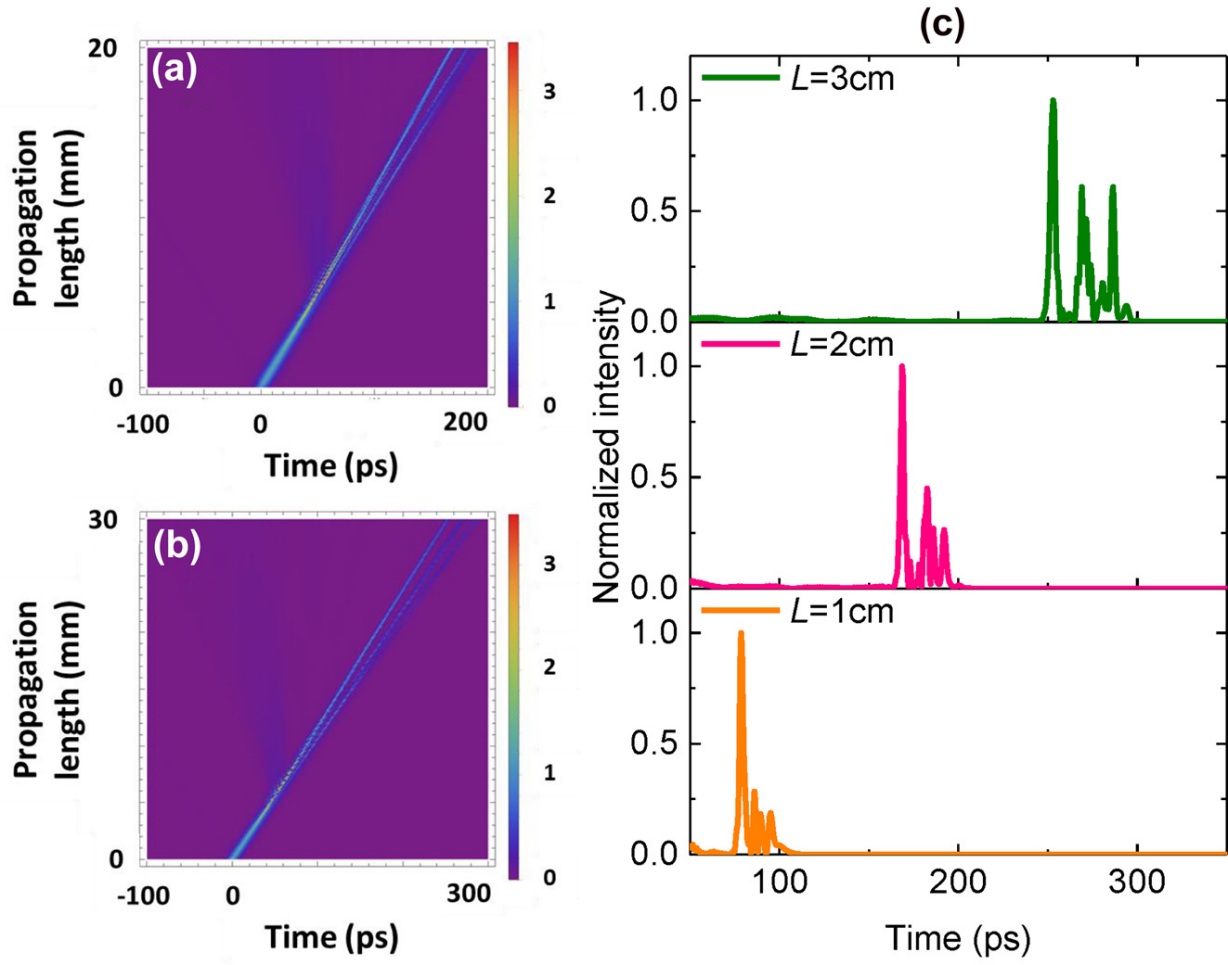
Evolution of temporal gap soliton along propagation length of 20 mm (a) and 30 mm, (b) and output temporal waveforms showing the temporal delays for different device lengths (c) for *P*_in_ = 36.6 W.

We further evaluate the pulse propagation dynamics for longer grating lengths of 20 mm and 30 mm for *P*_in_ = 36.6 W. Figure 7(a) and (b) shows the simulated gap soliton evolution over the longer grating lengths. The temporal profile of the pulse at the output of CMBG with lengths *L* = 1 cm, 2 cm and 3 cm are shown in Figure 7(c). It is observed that *T*_d_ is proportional to the total CMBG length. At a fixed input peak power, the group velocity, $\frac{L}{T_{d}}$ is expected to remain the same regardless of the device length. Therefore, a longer grating length facilitates acquisition of a longer temporal delay. We observe an increase in temporal separation between the main lobe of the pulse and its sidelobes for longer propagation lengths. This phenomenon is expected from light distortion arising from the combination of the soliton effect and the retarded group velocity. With an increase in grating length, an increase in temporal separation between the main lobe and side lobes of the gap soliton is observed, as a result of large group velocity in the photonic band edges.

Photothermal effects can provide a significant shift of the grating resonance which leads to an increase in transmission on input intensity [31], similar to the bandgap shift on Kerr effect. However, the photothermal effect occurs on a much longer timescale [32] than the Kerr effect (which is responsible for gap solitons). The thermal nonlinear effect comes into play only if the time scale of absorption is smaller than the order of magnitude of the pulse width. Heat generated by pulses can impact the subsequent pulse if heat generated by the pulse cannot be dissipated sufficiently before the next pulses arrives. The dissipation time associated with USRN is shorter than the pulse repetition of 50 ns [33]. Therefore, photothermal/thermal nonlinear effects would impart a negligible contribution to inducing the red-shift of the photonic bandgap given the experimental conditions, and cannot be the origin of the intensity-dependent soliton effect observed here.

This work may be compared with previous studies on soliton dynamics [30] as both exploit the same USRN platform. Reference [30] relies on the native waveguide dispersion for soliton formation, which in turn relies on the waveguide geometry and material dispersion. Conversely, gap solitons are soliton solutions which exist within the stopband of the grating, where the dispersion involved in the observed soliton phenomena is not that of the waveguide, but that induced by the grating structure. The magnitude of grating dispersion far exceeds that of a photonic waveguide, by two to three orders of magnitude. Whereas the soliton dynamics using the USRN waveguide showed a higher temporal compression of 8.7× at higher powers, the underlying mechanism is fundamentally different. The observations of gap soliton-based phenomena reported in this paper includes tuning of temporal delay/slow light as well as temporal compression. These arise because the USRN Bragg grating could facilitate obtaining the variation of a larger group index at the photonic band edges whereas the USRN waveguide possesses almost the same group index near 1550 nm. The properties in the Bragg grating structure allow for the implementation of temporal delay/slow light. Therefore, gap soliton dynamics would be more multifunctional in optical applications than waveguide-based soliton dynamics.

In this paper, the peak power used ranges from 15.7 W to 36.6 W, similar to the peak power utilized for nonlinear optical signal processing applications such as supercontinuum generation, soliton temporal compression, spectral broadening, and optical parametric amplification implemented in the USRN platform. However, when comparing with other CMOS photonics platforms such as stoichiometric silicon nitride, the peak power used for the realization of nonlinear photonic waveguide applications is significantly lower. For example, the generation of supercontinuum in stoichiometric silicon nitride typically requires peak powers on the order of kilowatts [34]. Note that the stoichiometric silicon nitride platform has no demonstrations of gap soliton phenomena. Therefore, we compare the peak powers to nonlinear applications such a supercontinuum generation. We note further that the peak powers used for the gap soliton effects are low as a result of the two orders of magnitude larger nonlinear parameter in USRN waveguides compared to stoichiometric silicon nitride waveguides. The peak powers used in this work require an average power of several milliwatts, which is easily accessed using conventional light sources and therefore do not pose a hindrance to adoption in applications. In addition, further reduction in the peak powers used for achieving the formation of gap solitons may be achieved by modal engineering of the USRN waveguide in the future. A reduction in the effective area of the USRN waveguide used to implement the grating could lead to reductions in the required peak power.

Slow light is a phenomenon that sparks immense curiosity amongst physicists. Beyond its intellectual merits, the ability to slow light holds tremendous promise in a variety of practical applications, including their use in optical buffers, optical delay lines, optical storage. Of these, optical storage is the most elusive of all, for compared to their electronic analogue where bits of electronic data can be easily stored using transistor states, the complete storage of bits of data in an optical vessel is extremely difficult. In the absence of true light storage, researchers have attempted to approach optical storage functions for example by using light to change the state of photosensitive materials as a way to represent bits. However, these do not represent true light storage, merely using light as a tool to switch between binary material states (crystalline and amorphous).

Kramers Kronig relations describe an intrinsic tradeoff between achievable bandwidth and group delay (dispersion), commonly referred to as the delay bandwidth product (DBP) [35]. As a consequence, there is an inverse relationship between the optical bandwidth and the achievable delay. For example, approaches for generating slow light such as Mach-Zehnder intensity (MZI) modulators suffer from the DBP limitation due to their reliance on a linear optical response [36]. For this reason, approaches that achieve the large DBP are active areas of research. Photonic crystal waveguides (PhCWs) are popular devices to generate slow light, through enhanced nonlinearities induced by the PhCW structure [37, 38]. However, despite the larger DBP of PhCWs compared to MZI modulators [39], generating slow light for use in tunable temporal delay and compression is not easily achieved because of the difficulty associated with precise control of dispersion compensation, leading to high net buffer capacity, a key factor on how much time of optical wave packets are stored and adjusted [40]. Notably, stimulated Brillouin scattering has been recently shown to be effective in achieving on-chip slow light [41]. As chip-scale miniaturization continues to be driven by both industry trends and advancements in CMOS manufacturing, novel methods with which to efficiently generate slow light on a chip are therefore topics which are of great interest for applications in future optical computing systems, all-optical information processing and data storage. The approach presented here could provide a new avenue for the generation of slow light as well as the tunable properties of delay on optical intensity. Importantly, it paves the way for a compact, low power, CMOS-chip scale solution towards optical buffers and optical storage.

## 3 Conclusions

In summary, we observe signatures of gap soliton propagation in a CMOS-compatible USRN Bragg grating. In our experiments, we demonstrate tunable slow light and temporal compression of picosecond pulses and intensity-dependent transmission due to self-switching of the photonic stopband. The experimentally observed change in the temporal delay of 7 ps agrees well with theory. In addition, 2.7× temporal compression is experimentally achieved for *P*_in_ of 15.7 W–36.6 W. We experimentally observe a 20% group velocity reduction compared to an USRN waveguide as well, corresponding to a slowing of light to 35% of the speed of light in vacuum for *P*_in_ of 15.7 W. The results showcase a new on-chip configuration for the generation of tunable slow light for possible future implementation for optical delay lines, optical buffers, potentially providing a pathway towards true optical storage on a chip.
